# Standards for conducting and reporting consensus and recommendation documents: European Society of Cardiovascular Radiology policy from the Guidelines Committee

**DOI:** 10.1186/s13244-024-01755-z

**Published:** 2024-08-14

**Authors:** Amalia Lupi, Dominika Suchá, Giulia Cundari, Nicola Fink, Hatem Alkadhi, Ricardo P. J. Budde, Federico Caobelli, Carlo N. De Cecco, Nicola Galea, Maja Hrabak-Paar, Christian Loewe, Julian Luetkens, Giuseppe Muscogiuri, Luigi Natale, Konstantin Nikolaou, Maja Pirnat, Luca Saba, Rodrigo Salgado, Michelle C. Williams, Bernd J. Wintersperger, Rozemarijn Vliegenthart, Marco Francone, Alessia Pepe

**Affiliations:** 1 Institute of Radiology, Department of Medicine—DIMED, University of Padua, Padua, Italy; 2 Department of Radiology and Nuclear Medicine, University Medical Center Utrecht, Utrecht, The Netherlands; 3 Department of Radiological, Oncological and Pathological Sciences, Sapienza University of Rome, Rome, Italy; 4 Department of Radiology, LMU University Hospital, LMU Munich, Munich, Germany; 5 Diagnostic and Interventional Radiology, University Hospital Zurich, University of Zurich, Zurich, Switzerland; 6 Department of Radiology and Nuclear Medicine, Erasmus Medical Center, Rotterdam, The Netherlands; 7 Department of Nuclear Medicine, University Hospital Bern and University of Bern, Bern, Switzerland; 8 Division of Cardiothoracic Imaging, Department of Radiology and Imaging Sciences, Emory University, Atlanta, GA USA; 9 Department of Diagnostic and Interventional Radiology, University Hospital Centre Zagreb, Zagreb, Croatia; 10 Division of Cardiovascular and Interventional Radiology, Department of Biomedical Imaging and Image-Guided Therapy, Medical University of Vienna, Vienna, Austria; 11 Department of Diagnostic and Interventional Radiology, University Hospital Bonn, Bonn, Germany; 12 Department of Radiology, ASST Papa Giovanni XXIII, Bergamo, Italy; 13 Department of Radiological Sciences—Institute of Radiology, Catholic University of Rome, “A. Gemelli” University Hospital, Rome, Italy; 14 Department of Diagnostic and Interventional Radiology, University of Tuebingen, Tübingen, Germany; 15 University Clinical Center Maribor, Maribor, Slovenia; 16 Radiology Department, AOU Cagliari, Policlinico Di Monserrato (CA), Monserrato, Italy; 17 Department of Radiology, Antwerp University Hospital, Edegem, Belgium; 18 Faculty of Medicine & Health Sciences, University of Antwerp, Edegem, Belgium; 19 Department of Radiology, Holy Heart Hospital, Lier, Belgium; 20 British Heart Foundation Centre for Cardiovascular Science, University of Edinburgh, Edinburgh, United Kingdom; 21 Department of Medical Imaging, University of Toronto, Toronto, ON Canada; 22 Peter Munk Cardiac Centre, Toronto General Hospital, University Medical Imaging Toronto, Toronto, ON Canada; 23 Department of Radiology, University of Groningen, University Medical Center Groningen, Groningen, Netherlands; 24 Department of Biomedical Sciences, Humanitas University via Rita Levi Montalcini 4, 20072 Pieve Emanuele Milan, Italy; 25 IRCCS Humanitas Research Hospital via Manzoni 56, 20089 Rozzano, Milan Italy

**Keywords:** Consensus, Cardiac imaging techniques, Radiology

## Abstract

**Abstract:**

Cardiovascular imaging is exponentially increasing in the diagnosis, risk stratification, and therapeutic management of patients with cardiovascular disease. The European Society of Cardiovascular Radiology (ESCR) is a non-profit scientific medical society dedicated to promoting and coordinating activities in cardiovascular imaging. The purpose of this paper, written by ESCR committees and Executive board members and approved by the ESCR Executive Board and Guidelines committee, is to codify a standardized approach to creating ESCR scientific documents. Indeed, consensus development methods must be adopted to ensure transparent decision-making that optimizes national and global health and reaches a certain scientific credibility. ESCR consensus documents developed based on a rigorous methodology will improve their scientific impact on the management of patients with cardiac involvement.

**Critical relevance statement:**

This document aims to codify the methodology for producing consensus documents of the ESCR. These ESCR indications will broaden the scientific quality and credibility of further publications and, consequently, the impact on the diagnostic management of patients with cardiac involvement.

**Key Points:**

- Cardiovascular imaging is exponentially increasing for diagnosis, risk stratification, and therapeutic management.
- The ESCR is committed to promoting cardiovascular imaging.
- A rigorous methodology for ESCR consensus documents will improve their scientific impact.

## Background and objective

The European Society of Cardiovascular Radiology (ESCR) is a non-profit scientific medical society dedicated to promoting and coordinating activities in cardiovascular imaging [1]. Imaging plays a strategic role in the diagnosis, risk stratification, and therapeutic management of patients with cardiovascular disease. Moreover, the current clinical guidelines drive an exponentially increasing demand for non-invasive cardiovascular imaging [2].

Various scientific documents have been developed or endorsed so far by the ESCR to present recommendations or expert consensus on specific topics [3–6]. Moreover, the ESCR is called upon to produce or endorse the following scientific documents according to the level of evidence provided:

- Clinical practice guidelines: evidence-based recommendations, based on a systematic review of the literature as assessed by expert panels and organizations, to offer standard approaches to clinical decision-making;
- Consensus documents: agreement or the consensus between experts on a specific topic based on critical appraisal of the literature together with expert opinion;
- Position papers on a specific issue, based on expert consensus without requiring a systematic review of the literature, to guide decision-making or inform the public; position papers provide expert recommendations on evolving issues, often based on limited evidence, and may be initiated by a specific group;
- White papers present a problem or explore a specific topic, providing an in-depth issue analysis, proposing an innovative approach, or discussing the implications of a new diagnostic or therapeutic approach. White papers aim to share ESCR’s opinion on a specific topic and should report and discuss the best available evidence.

Position and white papers follow the same preparation and approval procedures as for consensus documents, although a systematic literature review is not mandatory.

ESCR devised a standardized approach to creating such documents to streamline the process and avoid significant drawbacks, like the dominance of specific individuals, scientifically unsupported choices by strong opinions, and oversight of complex issues due to an unstructured process. Consensus development methods must be adopted to ensure transparent decision-making that optimizes national and global health and reaches a certain scientific credibility. Of note, reaching a formal consensus is established by utilizing already available information rather than meaning that the knowledge on a specific topic is implemented [7, 8].

The purpose of this document, written by ESCR committees and Executive board members and approved by the ESCR Executive Board and Guidelines committee, is to codify this standardized approach and detail the general rules for compiling and writing ESCR scientific documents.

Clinical practical guidelines are often also developed by clinicians, but the ESCR, through its delegates, could be called to endorse this type of document; thus, if imaging indications, techniques, or interpretation are part of a guideline, the inclusion of radiologists in the writing group and the application of the following indications is strongly advised. The following approach can also be applied to position and white papers except for a systematic literature search and review (SLR). Nevertheless, the ESCR highly recommends consensus documents as they align closely with the most stringent types of scientific literature utilized in radiology.

The target audience of this document is identified in guidelines committees, writing groups, collaborating organizations, and staff involved in developing scientific documents on cardiovascular imaging.

### Methodology

**Fig. 1:**
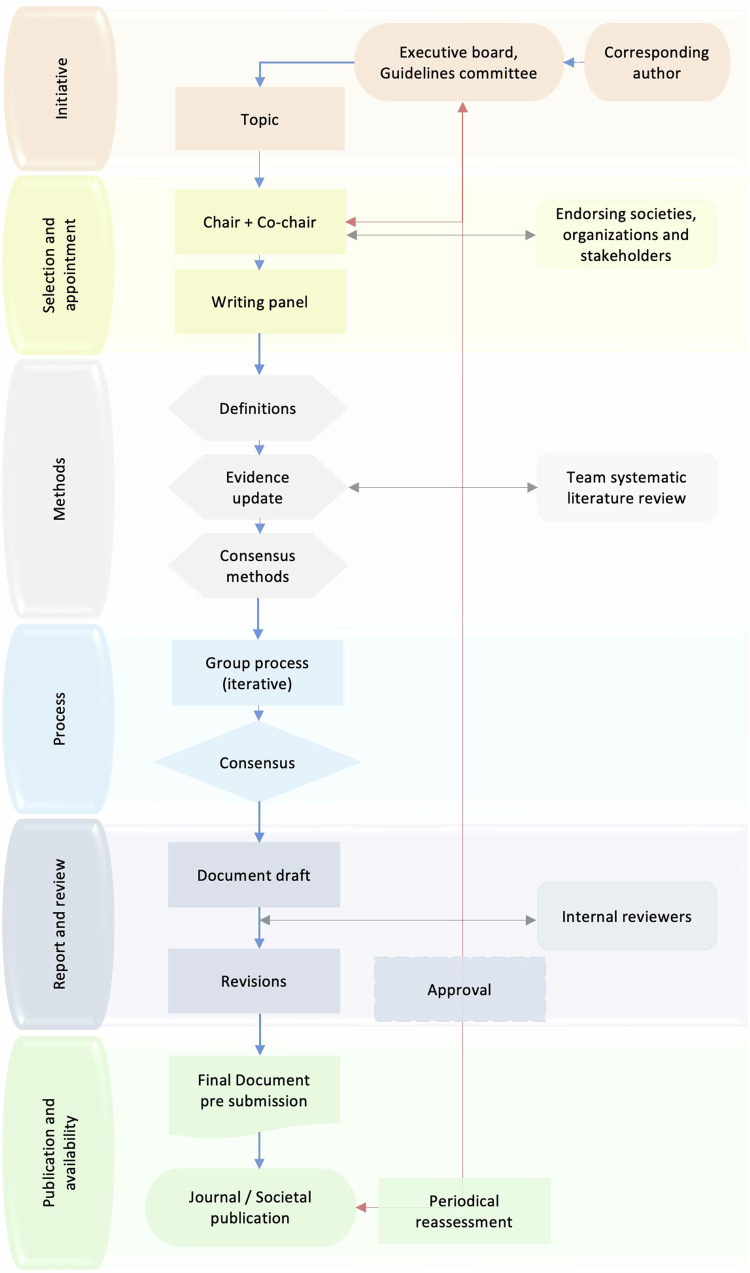
Overview of the steps for ESCR scientific documents

There may be unsolicited applications from an author or solicited by the ESCR Executive Board or Guideline Committee for a specific person to lead a writing group. The chair (corresponding author) submits the topic proposal to the ESCR Guidelines Committee through a specific form (office@escr.org) (Fig. 1). The ESCR Guidelines Committee evaluates the proposal and reserves the right to integrate it to guarantee full compliance with the current scientific objectives of the ESCR and the maximum clinical impact.

The writing group members are required to declare any conflicts of interest (COI).

### Panel selection and composition

The selection of a core group of panel members is crucial as the panel’s composition may significantly impact the document results. Member characteristics and experience determine their preferences and likely determine consensus ratings. Heterogenous panels have been shown to provide more diversity in opinions, knowledge, and perspectives and perform better than homogenous groups [9, 10]. Panel members are typically selected because of their knowledge and experience, stakeholder group representation (e.g., physicians, researchers, multidisciplinary, steering committee), affiliation to a specific profession, current board memberships, being a recognized authority in the field, or their willingness to participate [11]. Heterogeneity should also be sought regarding age, gender, region, and socio-economic background. Moreover, the involvement as co-authors of a patient indicated by a representative patients’ organization is strongly recommended, if applicable. Furthermore, including an evidence-based medicine specialist to guarantee a rigorous methodology is advisable.

No specific criteria exist for defining an ‘expert’, and the experience of panel members often remains unreported. Nevertheless, selection criteria should be predefined, the reasons behind such requirements should be clear and transparent and the selection process should be documented comprehensively. Including societies or specific individuals from a society in the panel is advised. COI of panel members should be defined, reported, and appropriately managed [12]. The involvement of stakeholders should be addressed when not being a part of the expert panel [12].

The number of panel members depends on the chosen consensus methodology. It typically includes a minimum of five to a hundred with Delphi and ‘modified’ Delphi methods, and this number can vary per round. In contrast, five to ten panelists seem optimal based on the nominal group technique (NGT). Combinations of consensus methods, the so-called multi-methods, are also gaining popularity and will be discussed below [13].

The selection and tasks of the external review group will be discussed at the end of this document. Based on the need for a multidisciplinary patient-centered approach, it is encouraged to invite other societies and organizations for formal endorsement to spread the impact of the document further.

The ESCR strongly recommended applying the following criteria in the panel selection and composition:

- proven expertise in the field: H-index > 15 for authors ≥ 40 years, H-index > 8 for authors < 40 years old, with at least two publications in the specific field of the document,
- gender balance: at least 30% of representation for each category (male/female),
- age balance: at least 30% of representation for each category (≥ 40/< 40 years old),
- Economic geographic balance: at least one representative from upper-middle, lower-middle- or low-income country groups [https://datahelpdesk.worldbank.org/knowledgebase/articles/906519-world-bank-country-and-lending-groups].

The chair will be the one to apply the topic proposal to the ESCR Guideline Committee.

The chair (corresponding author) will also indicate the co-chair (first author/last author). The chair is required to have an H-index > 25 and at least five publications on the specific topic. Nevertheless, the ESCR Guideline Committee has the right to evaluate the proposal and proposed panel members and propose adaptations.

### Procedure of the writing group

The panel group is encouraged to organize a brainstorming session to clearly define the specific clinical question addressed in the scientific document. This is important, as a straightforward question will ascertain finding an answer and increase the results’ reliability. The objective and rationale for the consensus process should be clear. The population to which the clinical problem applies should be clearly defined [14]. Predefining the consensus document’s target audience and potential users is essential, as its content should be adjusted accordingly. It is also recommended to define expected results and their use and impact on this audience. Last, the panel should also specify all outcomes that may be relevant for clinical decision-making.

Expert panel members must be current with current guidelines, standards, and recent publications to achieve reliable consensus results. Hence, all expert panel group members should receive a literature-based summary on the specific topic or clinical problem, as discussed in the following section.

### Evidence

A systematic literature search and review (SLR) is recommended to support reported scientific evidence (Fig. 1) [15].

The SLR on the specific issue could be an independent preliminary publication or addendum/summary as part of the main document, coordinated by two authors of the primary scientific document and up to six young researchers (< 40 years old) gender balanced. A PhD degree or a PhD training (or equivalent formal demonstration of scientific/academic work excellence) is considered a qualifying criterion to be selected in the SLR as a young researcher. Moreover, young researchers did not necessarily need to be involved in the primary scientific document.

Keywords should be carefully defined by the writing group and the search strategy described in the document, as well as any language or time interval restriction applied. An Evidence-Based Medicine specialist could be consulted to ensure the correct search methodology.

The proposed approach involves two independent young investigators using two or three databases (e.g., PubMed, Scopus, Web of Science). A citation manager (e.g., Mendeley, Endnote) could be used to collect and manage all results and automatically remove duplication. An automatic email search alert to identify newly published studies could be set up for each database.

Eligibility criteria should be defined and described in the document.

Up to six young researchers, in couples, proceed with SLR by screening the titles and abstracts of the records. The full text of the documents considered eligible is then evaluated for data extraction; any disagreement should be resolved by consensus, and cross-review on a limited sample should be considered to establish consistency.

For data synthesis, the best methodological/statistical practice should be followed.

### Consensus development and definition

The choice of the method for developing the consensus is critical to producing documents that effectively impact the clinical management of the patients. Several methods are available such as consensus development panel or conferences, NGT, Delphi, and RAND/UCLA appropriateness method.

The ESCR recommends using NGT or Delphi to represent a good balance between rigor and feasibility, particularly considering the specific imaging field.

NGT will be used to reach a formal consensus [13]. NGT has the advantage of allowing for the generation of more ideas and the possibility of discussing ideas among the participants. Face-to-face meetings could also be held online using common platforms.

NGT will be articulated in the following stages: (1) preparation and presentation of the items to be discussed; (2) silent idea generation (each member independently generates ideas or solutions to the topic); (3) sharing ideas in a round-robin fashion (one idea at a time); (4) clarification and discussion (each idea is debated in order every member understands it); (5) private ranking; (6) feedback to the participants. If consensus is not reached, a further discussion and private voting session can be carried out. A mediator should be present for each group to ensure every participant can share opinions and vote.

A Delphi method to reach a formal consensus can be adopted as well [13]. Compared to NGT, Delphi has the advantage of ensuring the anonymity of participants (they never meet or interact), enabling balanced ideas among panelists, reducing individual bias, and including a higher number of participants [16]. Also, for Delphi, we have different stages: (1) identification of the topic to be discussed; (2) literature research; (3) preparation of the questionnaire; (4) iterative round of responses (answers are always anonymized and shared with other participants); (5) individual or group feedback between rounds; (6) final report. A facilitator must be present to ensure anonymity.

Feedback to participants after each round should be given as a statistical analysis (in the case of quantitative parameters), including new or modified items. There will be no need for further discussion in case of immediate agreement on a specific item, and it should be removed from the next rounds [11].

The ranking should be given using a 9-point Likert scale [11]. Reaching consensus is defined when ≥ 70% of participants converge on a score ≥ 7/9 [11, 13]. When using the Delphi method, the iterative process is restarted until the predefined threshold for consensus is reached or three rounds are completed [17].

### Reporting

Appropriate and transparent reporting of writing group and expert panel processes holds similar importance to that of reporting scientific documents. The report provides the reader with information on the quality of the approach, content, conclusions, and generalizability to other populations or settings. Unfortunately, systematic literature studies have shown a lack of appropriate reporting of consensus building [10, 11, 13, 18, 19]. The EQUATOR (enhancing the quality and transparency of health research) Network aims to improve reporting quality. However, guidelines for reporting consensus documents have not been available. (https://www.equator-network.org/) Recently, van Zuuren et al presented a potential checklist outlining elements to integrate into guidelines on reporting consensus papers [18]. Their guideline project (ACCORD; accurate consensus reporting document) has just been published [20].

### Reporting of ESCR consensus documents

Reports of consensus projects should adhere to a structured and standardized format to ensure a comprehensive and transparent description of each step and to provide an explanation for the decisions taken, thereby adding credibility, and reducing bias. In Table 1, such a structured and standardized format is provided as a checklist. In short, (1) the rationale and aim of the consensus document should be clearly defined, (2) the selection, appointment, and background of the expert panelists need to be reported, (3) PRISMA 2020 (Preferred reporting items for systematic reviews and meta-analyses) guidelines [15] should be followed, (4) consensus methods and consensus process and decisions should be thoroughly reported to ensure reproducibility, including a clear definition of consensus (5) it is encouraged to report results on both the consensus process and its content, (6) study results and (non)-consensus need to be interpreted and put in perspective (7) the report should include a discussion of the strengths and limitations and their impact on applicability and generalizability. The inclusion of figures such as flow diagrams, tables, and supplemental material is highly recommended to enhance transparency and efficacy and provide further insights. Standardized terminology is recommended to minimize disparities and misinterpretation [21, 22]. When standardized terminology is lacking, further, more detailed specification is required. This is especially important for the term “modified” Delphi, as various methodological adaptations have been grouped under this label [11, 23].

**Table 1:** Format for structured and standardized reporting of consensus documents

Paragraph	Subsection	Content^a^
Introduction	Background	General introduction to the topic, current setting, guidelines
	Rationale	
	Objective	May encompass incorporation in a guideline
Participants	Panel	Selection process and rationale
		Composition, background, qualifications
		Potential conflicts-of-interest
		Role of the steering committee
	External reviewers	Composition
		Tasks/Goals
		Potential conflicts-of-interest
	Endorsement	Societies
	Stakeholders	
Process	Clinical problem	Precise definition
		Objective and rationale
		Target population
		Target audience
	Available evidence	Literature review and appraisal following PRISMA
		Method for presentation of literature to panel
	Consensus method^b^	Rationale
		Consensus method type
		Selected items/questionnaire/pretesting
		Voting rounds
		Stopping criteria
		Feedback/iteration (time points, content, distribution method)
		Interim evaluation and dealing with decisions (non-consensus)
		Data synthesis (method)
		Anonymity^c^
	Consensus	Definition (a priori)
		Levels
Results	Process	Literature search
		Panel response/drop out
		Items final/dropped
	Consensus	Outcomes
		Level of evidence
Discussion	Discussion	Strengths and limitations
		Applicability and generalizability
Conclusion	Conclusion	

^a^ Content that should be reported at a minimum but not restricted to

^b^ A flowchart or table outlining each step of the consensus method process is encouraged

^c^ Recommended if applicable

### Internal review

The internal peer review process ensures scientific research consensus documents’ quality, reliability, and credibility. To maintain objectivity, this review process should be performed by ESCR and should include three to five peer reviewers. The chair of the writing group chooses reviewers according to the chairperson of the Guidelines Committee.

As a first step, it is essential to select suitable reviewers, considering the following points: (1) Expertise: internal reviewers should be experts in the same or at least a closely related field as the consensus topic, with a broad knowledge of the subject matter, including relevant methods and current standards. The selection regarding a reviewer’s expertise should also consider their scientific impact. Thus, an H-index > 15 with at least three publications in the specific field of the document is recommended. (2) Diversity: ideally, external reviewers should have diverse backgrounds (sex, age, economic geographic area, and multidisciplinarity) to ensure a comprehensive assessment and reduce bias. (3) Independence: COI with the authors of the consensus document should be avoided to guarantee an independent external review process.

In addition to independent internal reviewers, recommendations from the editorial board can also be sought.

All internal reviewers should receive clear instructions on reviewing the consensus document. These criteria should include aspects such as appropriateness of the panel selection, clarity of consensus, validity of scientific methods, topic-related evidence, rigor of conclusions, and adherence to ethical standards.

All comments from internal peer reviewers must be seriously considered. When the scientific document is published, peer reviewer names and disclosures will also be published with the scientific document.

Once submitted to a scientific journal for dissemination, the document will be reviewed as foreseen in the regular journal’s evaluation process.

## Conclusions—GRADE/interpretation and SWOT analysis

Since consensus documents aim to provide recommendations by synthesizing diverse viewpoints and establishing a shared agreement among experts within a specific field, it is essential to include a compelling conclusion at the end of the document. Based on the document’s goal and target audience, this conclusion should summarize the key findings, highlight implications, and discuss strengths and limitations.

To clearly understand the recommendations’ significance, it is recommended to rate the certainty of evidence transparently. This should preferably be done using the GRADE (grading of recommendations assessment, development, and evaluation) system [24–26], as it reduces the multiplicity of conflicting systems for grading evidence and recommendations by unifying several aspects into a single system. It is a systematic approach to assess the quality of evidence and the strength of recommendations, classified into four and two levels, respectively. The levels of evidence are “high”, “moderate”, “low”, and “very low”. Recommendations are either defined as “strong” (benefits outweigh the risks) or “weak/conditional” (the balance between benefits and risks is less confident). This can help to provide a clear and concise summary of the main findings.

In addition to summarizing the key findings, discussing strengths, weaknesses, opportunities, and threats (SWOT) related to the consensus topic is crucial. This approach provides a comprehensive assessment of consensus recommendations by highlighting positive aspects (strengths and opportunities) while identifying potential challenges (weaknesses and threats) that may hinder implementation.

### Publication/availability/updates

The scientific documents will be preferentially submitted to the official reference societies journals, which will be applied for an external review as previously described. The publications should be open source and/or available on the official ESCR website. When necessary, the ESCR Guidelines Committee reviews scientific documents based on the opinion of Committee members or the presence of new evidence.

The Guidelines Committee reviews scientific documents at least every two years after publication to verify their currency and validity and eventually proposes an update to optimize patient management (Fig. 1).

## Abbreviations

COI: Conflict of interest
ESCR: European Society of Cardiovascular Radiology
NGT: Nominal group technique

## References

[1] Natale L, Vliegenthart R, Salgado R et al (2023) Cardiac radiology in Europe: status and vision by the European Society of Cardiovascular Radiology (ESCR) and the European Society of Radiology (ESR). Eur Radiol 33:5489–5497. 10.1007/s00330-023-09533-z 36905466 10.1007/s00330-023-09533-z PMC10006558

[2] Arbelo E, Protonotarios A, Gimeno JR et al (2023) ESC guidelines for the management of cardiomyopathies. Eur Heart J 44:3503–3626. 10.1093/eurheartj/ehad194 37622657 10.1093/eurheartj/ehad194

[3] Francone M, Budde RPJ, Bremerich J et al (2020) CT and MR imaging prior to transcatheter aortic valve implantation: standardisation of scanning protocols, measurements and reporting-a consensus document by the European Society of Cardiovascular Radiology (ESCR). Eur Radiol 30:2627–2650. 10.1007/s00330-019-06357-8 31489471 10.1007/s00330-019-06357-8 PMC7160220

[4] Francone M, Gimelli A, Budde RPJ et al (2022) Radiation safety for cardiovascular computed tomography imaging in paediatric cardiology: a joint expert consensus document of the EACVI, ESCR, AEPC, and ESPR. Eur Heart J Cardiovasc Imaging 23:e279–e289. 10.1093/ehjci/jeac048 35262687 10.1093/ehjci/jeac048

[5] Saba L, Loewe C, Weikert T et al (2023) State-of-the-art CT and MR imaging and assessment of atherosclerotic carotid artery disease: standardization of scanning protocols and measurements-a consensus document by the European Society of Cardiovascular Radiology (ESCR). Eur Radiol 33:1063–1087. 10.1007/s00330-022-09024-7 36194267 10.1007/s00330-022-09024-7 PMC9889495

[6] Saba L, Loewe C, Weikert T et al (2023) State-of-the-art CT and MR imaging and assessment of atherosclerotic carotid artery disease: the reporting-a consensus document by the European Society of Cardiovascular Radiology (ESCR). Eur Radiol 33:1088–1101. 10.1007/s00330-022-09025-6 36194266 10.1007/s00330-022-09025-6 PMC9889425

[7] Murphy MK, Black NA, Lamping DL et al (1998) Consensus development methods, and their use in clinical guideline development. Health Technol Assess 2:1–88 9561895

[8] Arakawa N, Bader LR (2022) Consensus development methods: considerations for national and global frameworks and policy development. Res Social Adm Pharm 18:2222–2229. 10.1016/j.sapharm.2021.06.024 34247949 10.1016/j.sapharm.2021.06.024

[9] Hauer KE, Cate OT, Boscardin CK et al (2016) Ensuring resident competence: a narrative review of the literature on group decision making to inform the work of clinical competency committees. J Grad Med Educ 8:156–164. 10.4300/JGME-D-15-00144.1 27168881 10.4300/JGME-D-15-00144.1 PMC4857505

[10] Boulkedid R, Abdoul H, Loustau M et al (2011) Using and reporting the Delphi method for selecting healthcare quality indicators: a systematic review. PLoS One 6:e20476. 10.1371/journal.pone.0020476 21694759 10.1371/journal.pone.0020476 PMC3111406

[11] Jünger S, Payne SA, Brine J et al (2017) Guidance on conducting and reporting Delphi studies (CREDES) in palliative care: recommendations based on a methodological systematic review. Palliat Med 31:684–706. 10.1177/0269216317690685 28190381 10.1177/0269216317690685

[12] Schünemann HJ, Wiercioch W, Etxeandia I et al (2014) Guidelines 2.0: systematic development of a comprehensive checklist for a successful guideline enterprise. CMAJ 186:E123–E142. 10.1503/cmaj.131237 24344144 10.1503/cmaj.131237 PMC3928232

[13] Humphrey-Murto S, Varpio L, Wood TJ et al (2017) The use of the Delphi and other consensus group methods in medical education research: a review. Acad Med 92:1491–1498. 10.1097/ACM.0000000000001812 28678098 10.1097/ACM.0000000000001812

[14] Brouwers MC, Kho ME, Browman GP et al (2010) AGREE II: advancing guideline development, reporting and evaluation in health care. CMAJ 182:E839–E842. 10.1503/cmaj.090449 20603348 10.1503/cmaj.090449 PMC3001530

[15] Page MJ, McKenzie JE, Bossuyt PM et al (2021) The PRISMA 2020 statement: an updated guideline for reporting systematic reviews. BMJ 372:n71. 10.1136/bmj.n71 33782057 10.1136/bmj.n71 PMC8005924

[16] Khodyakov D, Grant S, Kroger J, Gadwah-Meaden C, Motala A, Larkin J (2023) Disciplinary trends in the use of the Delphi method: a bibliometric analysis. PLoS One 18:e0289009. 10.1371/journal.pone.0289009 37582076 10.1371/journal.pone.0289009 PMC10427003

[17] Foth T, Efstathiou N, Vanderspank-Wright B et al (2016) The use of Delphi and nominal group technique in nursing education: a review. Int J Nurs Stud 60:112–120. 10.1016/j.ijnurstu.2016.04.015 27297373 10.1016/j.ijnurstu.2016.04.015

[18] van Zuuren EJ, Logullo P, Price A, Fedorowicz Z, Hughes EL, Gattrell WT (2022) Existing guidance on reporting of consensus methodology: a systematic review to inform ACCORD guideline development. BMJ Open 12:e065154. 10.1136/bmjopen-2022-065154 36201247 10.1136/bmjopen-2022-065154 PMC9462098

[19] Sinha IP, Smyth RL, Williamson PR (2011) Using the Delphi technique to determine which outcomes to measure in clinical trials: recommendations for the future based on a systematic review of existing studies. PLoS Med 8:e1000393. 10.1371/journal.pmed.1000393 21283604 10.1371/journal.pmed.1000393 PMC3026691

[20] Gattrell WT, Logullo P, van Zuuren EJ et al (2023) ACCORD (accurate consensus reporting document): a reporting guideline for consensus methods in biomedicine developed via a modified Delphi. Plos Med 21:e1004326. 10.1371/journal.pmed.1004326 10.1371/journal.pmed.1004326 PMC10805282 38261576

[21] Koweek L, Achenbach S, Berman DS et al (2023) Standardized medical terminology for cardiac computed tomography 2023 update: an expert consensus document of the Society of Cardiovascular Computed Tomography (SCCT), American Association of Physicists in Medicine (AAPM), American College of Radiology (ACR), North American Society for Cardiovascular Imaging (NASCI) and Radiological Society of North America (RSNA) with endorsement by the Asian Society of Cardiovascular Imaging (ASCI), the European Association of Cardiovascular Imaging (EACI), and the European Society of Cardiovascular Radiology (ESCR). J Cardiovasc Comput Tomogr 5:e230167. 10.1016/j.jcct.2023.06.002 10.1016/j.jcct.2023.06.002 37495455

[22] Hundley WG, Bluemke DA, Bogaert J et al (2022) Society for Cardiovascular Magnetic Resonance (SCMR) guidelines for reporting cardiovascular magnetic resonance examinations. J Cardiovasc Magn Reson 24:29. 10.1186/s12968-021-00827-z 35484555 10.1186/s12968-021-00827-z PMC9052489

[23] Keeney S, Hasson F, McKenna H (2006) Consulting the oracle: ten lessons from using the Delphi technique in nursing research. J Adv Nurs 53:205–212. 10.1111/j.1365-2648.2006.03716.x 16422719 10.1111/j.1365-2648.2006.03716.x

[24] Guyatt GH, Oxman AD, Vist GE et al (2008) GRADE: an emerging consensus on rating quality of evidence and strength of recommendations. BMJ 336:924–336. 10.1136/bmj.39489.470347.AD 18436948 10.1136/bmj.39489.470347.AD PMC2335261

[25] Guyatt GH, Oxman AD, Kunz R et al (2008) What is “quality of evidence” and why is it important to clinicians? BMJ 336:995–998. 10.1136/bmj.39490.551019.BE 18456631 10.1136/bmj.39490.551019.BE PMC2364804

[26] Guyatt G, Oxman AD, Akl EA et al (2011) GRADE guidelines: 1. Introduction-GRADE evidence profiles and summary of findings tables. J Clin Epidemiol 64:383–394. 10.1016/j.jclinepi.2010.04.026 21195583 10.1016/j.jclinepi.2010.04.026

