# Correction: Standards for conducting and reporting consensus and recommendation documents: European Society of Cardiovascular Radiology policy from the Guidelines Committee

**DOI:** 10.1186/s13244-024-01811-8

**Published:** 2024-10-07

**Authors:** Amalia Lupi, Dominika Suchá, Giulia Cundari, Nicola Fink, Hatem Alkadhi, Ricardo P. J. Budde, Federico Caobelli, Carlo N. De Cecco, Nicola Galea, Maja Hrabak-Paar, Christian Loewe, Julian A. Luetkens, Giuseppe Muscogiuri, Luigi Natale, Konstantin Nikolaou, Maja Pirnat, Luca Saba, Rodrigo Salgado, Michelle C. Williams, Bernd J. Wintersperger, Rozemarijn Vliegenthart, Marco Francone, Alessia Pepe

**Affiliations:** 1 Department of Medicine—DIMED, Institute of Radiology, University of Padua, Padua, Italy; 2 Department of Radiology and Nuclear Medicine, University Medical Center Utrecht, Utrecht, The Netherlands; 3 Department of Radiological, Oncological and Pathological Sciences, Sapienza University of Rome, Rome, Italy; 4 Department of Radiology, LMU University Hospital, LMU Munich, Munich, Germany; 5 Diagnostic and Interventional Radiology, University Hospital Zurich, University of Zurich, Zurich, Switzerland; 6 Department of Radiology and Nuclear Medicine, Erasmus Medical Center, Rotterdam, The Netherlands; 7 Department of Nuclear Medicine, University Hospital Bern, University of Bern, Bern, Switzerland; 8 Division of Cardiothoracic Imaging, Department of Radiology and Imaging Sciences, Emory University, Atlanta, GA USA; 9 Department of Diagnostic and Interventional Radiology, University Hospital Centre Zagreb, Zagreb, Croatia; 10 Division of Cardiovascular and Interventional Radiology, Department of Biomedical Imaging and Image-Guided Therapy, Medical University of Vienna, Vienna, Austria; 11 Department of Diagnostic and Interventional Radiology, University Hospital Bonn, Bonn, Germany; 12 Department of Radiology, ASST Papa Giovanni XXIII, Bergamo, Italy; 13 Department of Radiological Sciences, Institute of Radiology, Catholic University of Rome, “A. Gemelli” University Hospital, Rome, Italy; 14 Department of Diagnostic and Interventional Radiology, University of Tuebingen, Tübingen, Germany; 15 University Clinical Center Maribor, Maribor, Slovenia; 16 Radiology Department, AOU Cagliari, Policlinico Di Monserrato (CA), Monserrato, Italy; 17 Department of Radiology, Antwerp University Hospital, Edegem, Belgium; 18 Faculty of Medicine & Health Sciences, University of Antwerp, Edegem, Belgium; 19 Department of Radiology, Holy Heart Hospital, Lier, Belgium; 20 British Heart Foundation Centre for Cardiovascular Science, University of Edinburgh, Edinburgh, UK; 21 Department of Medical Imaging, University of Toronto, Toronto, ON Canada; 22 Peter Munk Cardiac Centre, Toronto General Hospital, University Medical Imaging Toronto, Toronto, ON Canada; 23 Department of Radiology, University Medical Center Groningen, University of Groningen, Groningen, The Netherlands; 24 Department of Biomedical Sciences, Humanitas University, Milan, Italy; 25 IRCCS Humanitas Research Hospital, Rozzano, Italy

**Correction to: Insights into Imaging (2024) 15:207**

10.1186/s13244-024-01755-z

The publication of this article unfortunately contained mistakes. The given names of the authors were abbreviated by mistake. The complete names are given below.

Amalia Lupi^*1^, Dominika Suchá^*2^, Giulia Cundari^3^, Nicola Fink^4^, Hatem Alkadhi^5^, Ricardo P. J. Budde^6^, Federico Caobelli^7^, Carlo N. De Cecco^8^, Nicola Galea^3^, Maja Hrabak-Paar^9^, Christian Loewe^10^, Julian A. Luetkens^11^, Giuseppe Muscogiuri^12^, Luigi Natale^13^, Konstantin Nikolaou^14^, Maja Pirnat^15^, Luca Saba^16^, Rodrigo Salgado^17–19^, Michelle C. Williams^20^, Bernd J. Wintersperger^4,21,22^, Rozemarijn Vliegenthart^23^, Marco Francone^24,25^, Alessia Pepe^1^

The original article has been corrected.

